# Latent Diabetic Macular Edema in Chinese Diabetic Retinopathy Patients

**DOI:** 10.3389/fmed.2021.739656

**Published:** 2021-10-04

**Authors:** Xue Bai, Rui Hua

**Affiliations:** Department of Ophthalmology, First Hospital of China Medical University, Shenyang, China

**Keywords:** optical coherence tomography, diabetic retinopathy, fluorescein angiography, diabetic macular edema, central foveal thickness

## Abstract

**Purpose:** To compare the detection rates of optical coherence tomography (OCT) and fluorescein angiography (FA) in a diabetic macular edema (DME) and the severity of diabetic retinopathy in both color fundus images (CFI) and FA, and to investigate the predictive factors in macular leakages in FA.

**Methods:** This was a retrospective study, and a total of 132 eyes of 77 patients with diabetic retinopathy were enrolled. Macular OCT, FA, and CFI were reviewed and measured. Central foveal thickness was also measured.

**Results:** The severity of diabetic retinopathy in FA was significantly higher than that in CFI (*p* < 0.001). OCT detected 26 eyes with DMEs, which included the following: 13 eyes with cystoid macular edemas; 13 eyes with serous retinal detachments; 11 eyes with diffuse retinal thickening; 4 eyes with vitreomacular interface abnormalities. In contrast, 72 out of 132 eyes (54.5%) showed macular leakages in FA, which was significantly higher than that detected by OCT (*p* < 0.001). Compared with FA, the sensitivity and the specificity of OCT in detecting DMEs were 30.6 and 93.3%, respectively. However, central foveal thickness was not significantly different between the patients with non-clinically significant macular edema (CSME, 253.1 ± 26.95 μm) and slight CSME (270.9 ± 37.11 μm, *p* = 0.204). The mean central foveal thickness in diabetic macular edema (FA) eyes was 271.8 ± 66.02 μm, which was significantly higher than that (253. ± 25.21 μm) in non-DME (FA) eyes (*p* = 0.039). The central foveal thickness in DME (FA) eyes was significantly lower than that in eyes with DME (OCT) (*p* = 0.014). After adjusting for age and sex, a logistic regression analysis showed that the classification of diabetic retinopathy in FA was positively associated with macular leakage in FA (*p* < 0.001).

**Conclusions:** The severity of diabetic retinopathy is underestimated in CFI compared with that in FA. FA can detect latent DMEs, which appeared normal on OCT. The central foveal thickness is not a sensitive parameter for detecting latent DMEs.

## Introduction

A diabetic macular edema (DME) can occur at any stage of diabetic retinopathy (DR) and is the main cause for vision loss in patients with diabetes (1). In 2018, Song et al. reported that the pooled prevalences of any DR, non-proliferative DR (NPDR), and proliferative DR (PDR) were 1.14, 0.9, and 0.07%, respectively, in the general population *via* a meta-analysis (2). Jin et al. also reported that the 5-year DR incidence rate was 46.89% for 322 participants and more frequently occurred in patients with hyperglycemia and high blood pressure (3). Furthermore, Wang et al., in their Handan Eye Study, concluded that the prevalences of PDR, DME, and vision-threatening retinopathy were 1.6, 5.2, and 6.3%, respectively, in a rural population of northern China. However, DMEs are not unique to Chinese patients (4). In 2019, we reported that the prevalence of diabetic optic neuropathy in patients with Chinese DR was 38.4% [sample size: 1,067 eyes of 550 patients (5)]. Finally, we found that the late choroidal non-perfusion region is a risk factor in diabetic choroidopathy with DR (6).

Long-term hyperglycemia in patients with diabetes is attributed to the infiltration of plasma and liquid into the retinal tissue within the macula, which damages the blood-retinal barrier (BRB), resulting in retinal thickening and macular edemas (7). Hyperglycemia can initiate a series of linkage reactions, and various inflammatory factors and cytokines are upregulated. This leads to the loss of retinal pericytes, a damaged BRB, and enhanced permeability and leakage (8, 9). The pathological feature of a DME is its intraretinal or subretinal effusion in the macular area.

The common examinations for the clinical diagnosis of a macular edema include color fundus images (CFI), optical coherence tomography (OCT), and fundus angiography (FA). OCT is a non-invasive, accurate, and repeatable ophthalmological examination approach that can quantitatively measure retinal thickness, evaluate morphological changes, and analyze the characteristics of the structural hierarchy of the retina. The different morphologies of DMEs detected by OCT are classified into the following: diffuse retinal thickening (DRT), cystoid macular edema (CME), serous retinal detachment (SRD), and vitreomacular interface abnormality (VMIA) (10). FA can reflect the subtle structure of the fundus blood vessels and the dynamic changes of the retina, which are often difficult to capture by OCT. These changes in vascular structure and function can be evaluated by capturing the distribution morphology of fluorescein sodium after entering the ocular fundus vessels, which can provide a reference for discussing the pathogenesis and therapeutic effect of DR (11). The different morphologies of DMEs detected by FA are divided into focal macular edema, diffuse macular edema, and CME (12). Additionally, FA can detect the early stages of a DME in which light dispersion is seen around the microaneurysm, and the central macula is least affected (or not affected) by the exudate (13). Studies on DR blood flow density changes found that the foveal avascular zone (FAZ) of patients with DR accompanied by a DME was significantly larger than that of patients without DR accompanied by a DME (14). FA is also more specific, accurate, and subtle in understanding fundus blood vessels and retinas (15). In the guidelines for DR treatment, the difference between treatment regimens for patients with and without DME is indicated (16). Patients with mild-to-moderate NPDR with occasional spotted bleeding or hard exudates should be examined within 6–12 months. Patients with macular edema that is not clinically significant, should be examined within 3–4 months, since they are likely to develop a clinically significant macular edema (CSME) (17). However, we found that CFI, FA, and OCT results were inconsistent in some patients with DR. A DME is considered the most important cause of blindness in DR (18). We found that many patients with early NPDR had severe visual impairments due to a DME occurrence, although some lesions have been ignored. Therefore, we conducted a retrospective study to compare the detection rates of OCT and FA in DMEs, assess the severity of DR using both CFI and FA, and investigate the predictive factors in macular leakages in FA.

## Methods

This retrospective, cross-sectional, hospital-based study, which adhered to the tenets of the Declaration of Helsinki, was approved by the Institutional Review Board of China Medical University. Informed consent for their medical information to be included in this research was obtained.

### Study Subjects and Materials

**Figure 1:**
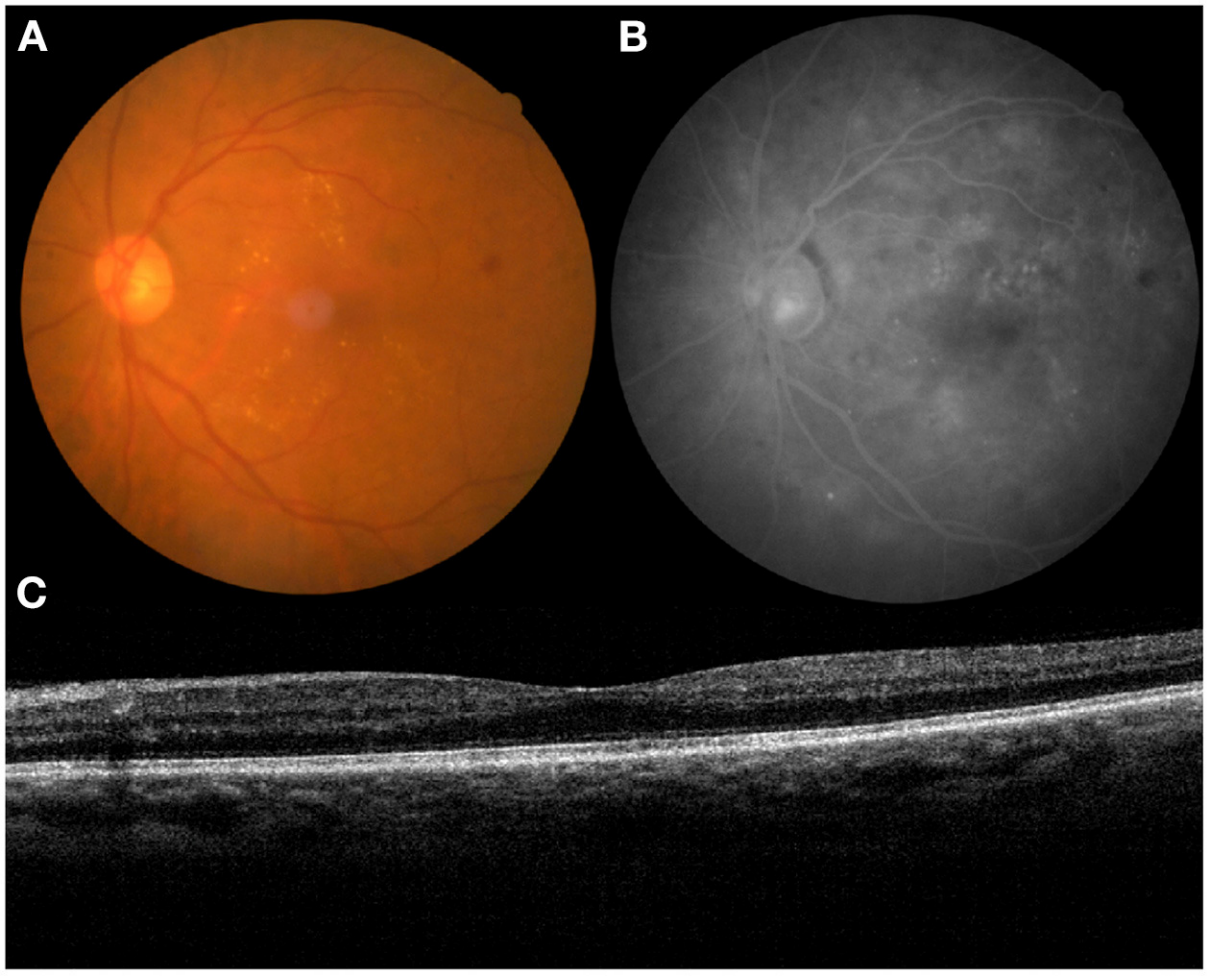
The medical record of a 63-year-old patient with diabetic retinopathy (DR) (left eye). **(A)** color fundus images (CFI), **(B)** fundus angiography (FA), and **(C)** optical coherence tomography (OCT).

The present study included patients with type 2 diabetes diagnosed with DR by CFI and FA, who referred to the First Hospital of China Medical University between January 2015 and December 2020. The medical records, including age, sex, CFI (Canon, CX-1, Tokyo, Japan), FA (Canon, CX-1, Tokyo, Japan), and OCT (Optovue, RTVue, Avanti, Optovue Inc., Fremont, CA, USA) scan results, were reviewed and measured. The scanning quality index of the OCT was above “60”. We identified the same 50° angle of the view between the CFI and FA (Figure 1).

Using the International Clinical Diabetic Retinopathy Disease Severity Scale (19), we evaluated the severity of DR in CFI and FA, which was also based on previous studies (20, 21). A DME was defined by hard exudates in the presence of microaneurysms and blot hemorrhages within one-disc diameter of the foveal center (1). Seven central horizontal lines of the OCT that were 6-mm length in their maculas were selected, and central foveal thickness (CFT) was automatically measured by a built-in software. The type of DME in the OCT images was assessed according to a previous study (10), where XB and RH accomplished the grading work together, including CFT, FA, and OCT. The patients who had poor quality images, other retinal diseases, or a history of ocular interventions (e.g., anti-vascular endothelial growth factor injection, laser photocoagulation, or vitrectomy in either eye) were excluded.

### Statistical Analyses

All statistical analyses were performed using the SPSS statistical software (version 18.0, SPSS, Inc., Chicago, IL, USA). Data are presented as mean ± standard deviation for variables with a normal distribution. The difference is the severity of DR in CFI and FA, and the different detection rates of DME in OCT and FA were analyzed using the Wilcoxon test. The sensitivity and the specificity of OCT in detecting DMEs were also calculated. We conducted a single-factor repeated measures analysis of variance (ANOVA) with a *post-hoc* least significance difference (LSD) test for pairwise comparisons to compare the CFT in the subtypes of CSME. The different CFTs in patients with non-DME and DME based on the OCT and FA results were compared using Student's *t*-test. Using multiple logistic regression analyses, the predictors of macular leakage in FA were examined after adjusting for age, sex, and the classification of DR in CFI and FA, CSME, DME in OCT, and CFT. The area under the receiver-operating characteristic (AUROC) curve was also plotted to test predictors for macular leakage in FA. Odds ratios (ORs) and 95% confidence intervals (CIs) were calculated. Statistical significance was defined as *p* < 0.05.

## Results

From 77 patients with DR, with the average age of 56.7 ± 9.65 years old, 132 eyes were finally analyzed, including 78 men and 54 women. Furthermore, 57 of 132 eyes were diagnosed with no DR (NDR, 43.2%), 7 eyes were diagnosed with mild NPDR (5.3%), 24 eyes were diagnosed with moderate NPDR (18.2%), 42 eyes were diagnosed with severe NPDR (31.8%), and 2 eyes were diagnosed with PDR (1.5%) according to CFI. In contrast, FA revealed NDR in 38 eyes, slight NPDR in 24 eyes, moderate NPDR in 22 eyes, severe NPDR in 14 eyes, and PDR in 34 eyes. The severity of DR assessed by FA was significantly higher than in CFI (*z* = 4.812, *p* < 0.001).

Additionally, there were 110 eyes without CSME, 10 eyes with slight CSME, 9 eyes with moderate CSME, and 3 eyes with severe CSME. Similarly, OCT detected 26 eyes with DME (19.7%, *z* = 1.736, *p* = 0.083), including 13 eyes with CME, 13 eyes with SRD, 11 eyes with DRT, and 4 eyes with VMIA.

In contrast, 72 out of 132 eyes (54.5%) showed macular leakages in FA, which was significantly higher than those detected on OCT (*z* = 3.85, *p* < 0.001). More eyes with CME (*n* = 31, *z* = 2.777, *p* = 0.005) and DRT (*n* = 39, *z* = 4.95, *p* < 0.001) were detected in the FA group than in the OCT group. In addition, only one eye with an inky leak was found in the FA group. Of 106 eyes without DME (OCT), macular leakages in 50 eyes were identified in FA. Compared with FA, the sensitivity and the specificity of OCT in detecting DMEs were 30.6 and 93.3%, respectively.

**Figure 2:**
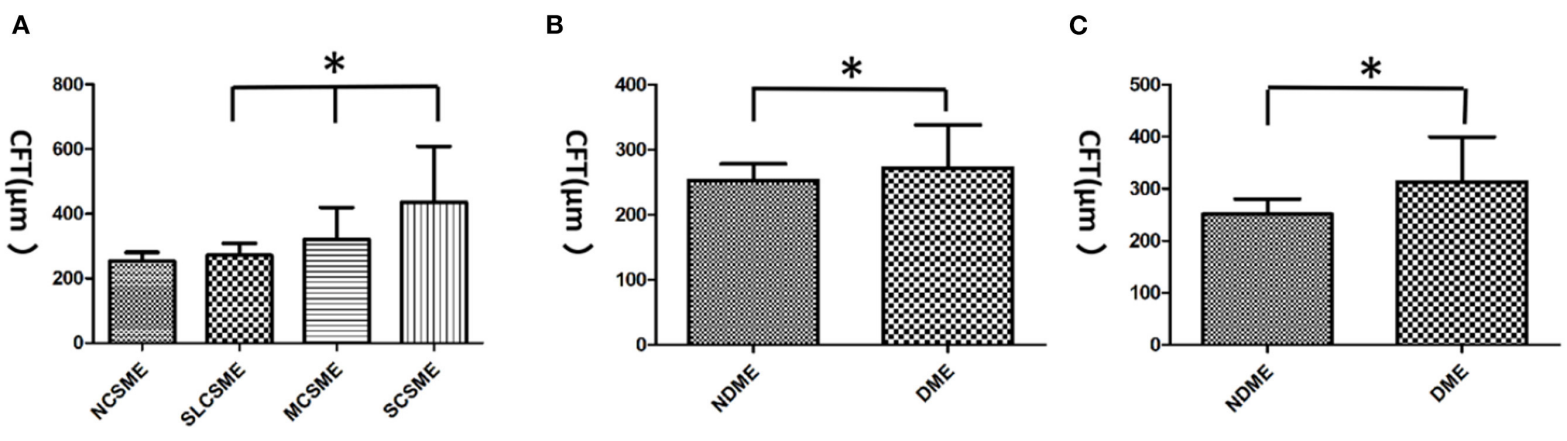
The central foveal thickness (CFT) in diabetic macular edemas (DMEs). **(A)** The CFT increased significantly from slight clinically significant macular edema (CSME) (270.9 ± 37.11 μm) to moderate CSME (320.8 ± 98.16 μm) and severe CSME (435.3 ± 172.28 μm). However, no difference was found between non-CSME (253.1 ± 26.95 μm) and slight CSME in CFT. **(B)** The mean CFT in DME (FA) eyes was 271.8 ± 66.02 μm, which was significantly higher than that (253. ± 25.21 μm) in non-DME (FA) eyes. **(C)** The mean CFT in DME (OCT) eyes was 312.8 ± 87.13 μm, which was significantly higher than that (251. ± 29.11 μm) in non-DME (OCT) eyes. *p < 0.05. NCSME, non-CSME; SLCSME, slight CSME; MCSME, moderate CSME; SCSME, severe CSME; NDME, non-DME.

The average CFT was 263.2 ± 52.32 μm. The CFT increased significantly from slight CSME (270.9 ± 37.11 μm) to moderate CSME (320.8 ± 98.16 μm, *p* = 0.011) and severe CSME (435.3 ± 172.28 μm, *p* < 0.001). However, the CFT (*p* = 0.204) was not different between non-CSME (253.1 ± 26.95 μm) and slight CSME (*p* = 0.204). The mean CFT in DME (OCT) eyes was 312.8 ± 87.13 μm, which was significantly higher than that (251.0 ± 29.11 μm) in non-DME (OCT) eyes (*t* = 6.099, *p* < 0.001). Similarly, the mean CFT in DME (FA) eyes was 271.8 ± 66.02 μm, which was significantly higher than that in non-DME (FA) eyes (253.0 ± 25 μm; *t* = 2.08, *p* = 0.039; Figure 2). Interestingly, the CFT in DME (FA) eyes was significantly lower than that in eyes with DME (OCT) (*t* = 2.491, *p* = 0.014).

Adjusted for age and sex, a logistic regression analysis showed that the classification of DR in FA was positively associated with macular leakage in FA [*B* = 1.82, Exp (*B*) = 0.162, *p* < 0.001]. However, the classification of DR in CFI showed a slightly negative relationship with macular leakage in FA [*B* = 0.8, Exp (*B*) = 2.225, *p* = 0.02].

**Figure 3:**
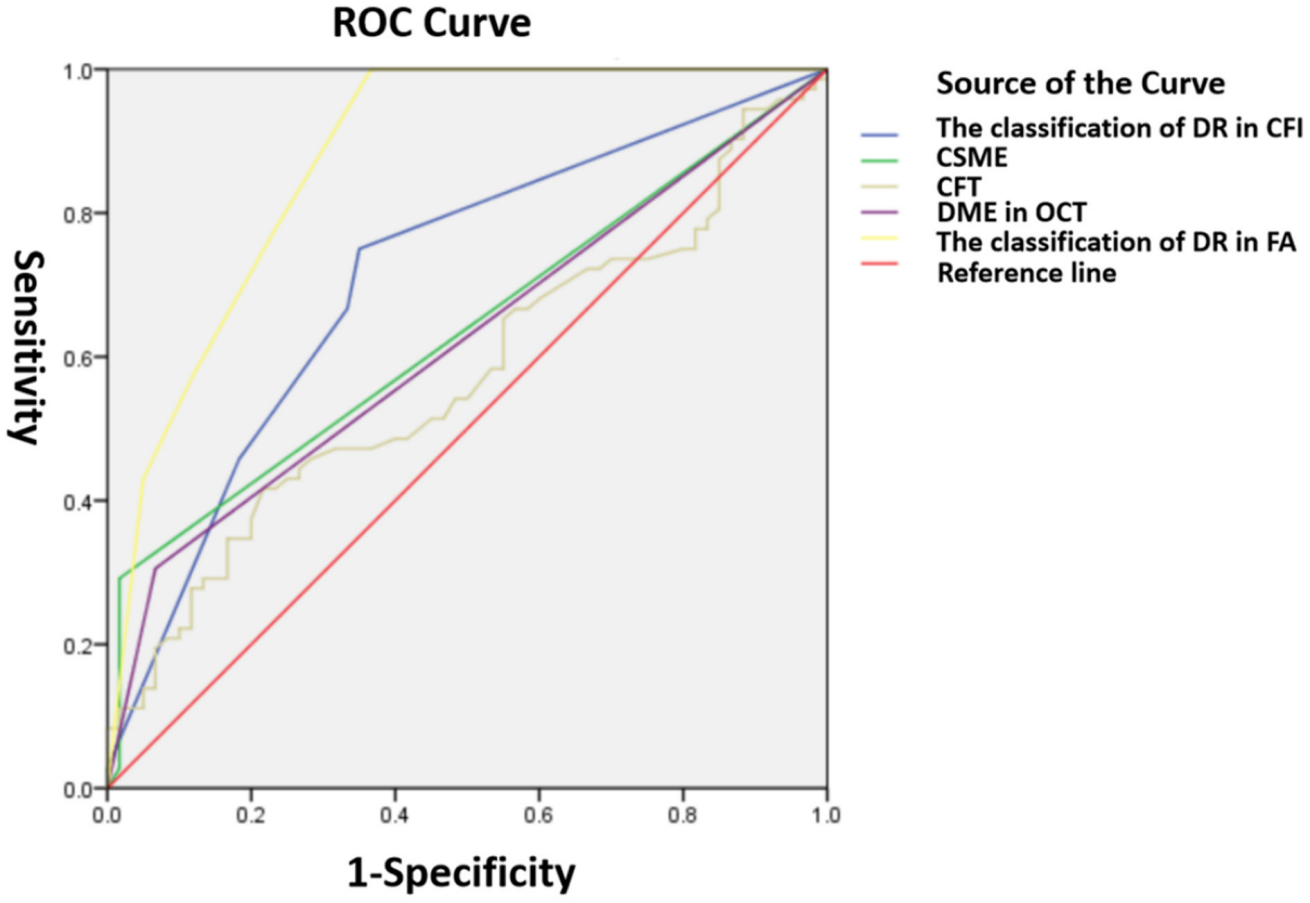
Receiver operating characteristic (ROC) curves for indicating macular leakages in FA. The classification of DR in CFI (0.709 ± 0.046), CSME (0.635 ± 0.048, *p* = 0.008), DME in OCT (0.619 ± 0.048), DR in FA (0.875 ± 0.031), and CFI (0.573 ± 0.05).

The results showed that the AUROC for the classification of DR in CFI [0.709 ± 0.046, *p* < 0.001, 95% CI (0.620, 0.799)], CSME [0.635 ± 0.048, *p* = 0.008, 95% CI (0.541, 0.729)], and DME [0.619 ± 0.048, *p* = 0.018, 95% CI (0.524, 0.714)] and the classification of DR in FA [0.875 ± 0.031, *p* < 0.001, 95% CI (0.813, 0.936)] significantly predicted macular leakages in FA, compared with CFI [0.573 ± 0.05, *p* = 0.152, 95% CI (0.475, 0.67); Figure 3].

## Discussion

In our study, the severity of DR assessed by FA was significantly higher than that in CFI. Hu et al. reported that the microcirculation of retinal vasculature could be dynamically observed in FA. The enhanced display of lesions can be obtained through a fluorescence contrast, which is conducive to judging the severity of hemangiomas, bleeding points, and macular edemas (22). In contrast, FA is also of great value to the identification of DR to different degrees. In the early stage of DR, CFI did not show bleeding and exudation, while FA showed a dye leakage. Recently, Soares et al. reported that an OCT angiography (OCTA) allows better discrimination of the central subfield and parafoveal macular microvasculature than FA, especially for FAZ disruption and capillary dropout, without the need for an intravenous injection of fluorescein (20). However, the comparison between FA and OCTA is only within a 3 × 3-mm region, and, to our knowledge, an OCTA cannot detect all types of DR lesions in contrast with both CFT and FA. Examples include retinal hemorrhage, hard exudates, cotton-wool spots, and vascular leakages. Importantly, the view of CFI and FA in our study was 50° using a Canon camera (CX-1), which can provide a more comprehensive assessment.

Our study found that the macular leakage in FA was significantly higher than in eyes with DME detected by OCT. More eyes with CME and DRT were detected in the FA group than in the OCT group. In contrast, Ouyang et al. reported that the sensitivity for detecting definite CME was higher for OCT (95%) than for FA (44%) (23). Compared with FA, the sensitivity and the specificity of OCT in detecting DME in our study were 30.6 and 93.3%, respectively. FA is commonly used for the diagnosis of DR at early stages. A DME can develop in any of the following conditions: if the damage to the BRB in the superficial blood vessels leads to the production of fluid exceeding discharge capacity; if the function of Müller cells is abnormal; or because the water transport disorder is caused by the damage of deep blood vessels (24). Therefore, at the early stages of a DME, FA images show a small leakage in the macular area when the BRB is initially destroyed, and there is no change in the retinal thickness in the macular area detected by OCT.

A CFT > 250 μm indicates a macular edema in OCT, and the CFT size of the eye of a healthy individual is 216.56 μm (95% CI 191.064 to 242.056 μm) (25). In our study, the CFT increased significantly from slight CSME (270.9 ± 37.11 μm) to moderate CSME (320.8 ± 98.16 μm) and severe CSME (435.3 ± 172.28 μm). Furthermore, CFT is an important parameter for assessing the severity of CME (26). The CFT in DME (FA) eyes was significantly lower than that in DME (OCT) eyes, and macular leakages developed before an increase in CFT. The results of FA showed that, in certain areas, cotton-wool spots and focal retinal capillary non-perfusion were observed before macular foveal thickness increased (27). However, the subtle structure and accompanying changes in FA are often the key factors affecting DME regression and determining the prognosis of visual function (28). We suggest that patients who present with the early stages of a macular leakage detected by FA be diagnosed with occult latent edemas to avoid treatment delays. We also considered that both early increased retinal capillary permeability and capillary dropout in the deep capillary plexus detected by OCTA contribute to latent DMEs.

The classification of DR in FA was positively associated with macular leakage in FA. However, the classification of DR in the CFI showed a slightly negative relationship with macular leakage in FA. In the DR classification, FA has high sensitivity and CFI has high specificity. Furthermore, CFI is a non-invasive examination measure with high safety and low cost and can be used for epidemiological screening of DR (29).

A major limitation of this study is the small sample size. Thus, in future studies, the sample size should be increased. Furthermore, we will consider the addition of more graders in future research.

In conclusion, this is the first report to compare the detection rate of DMEs by OCT and FA and assess the severity of DR by CFI and FA. We also identified the predictive factors of macular leakages in FA. The classification of DR in FA was positively associated with macular leakage in FA. Additionally, CFI underestimated the severity of DR compared with FA. The CFT is not a sensitive parameter for detecting latent DME. FA can detect latent DMEs, which appeared normal on OCT scans. Our results suggest that the early stages of macular leakage can be diagnosed as latent DMEs, which may be beneficial for the early diagnosis and treatment of DMEs and facilitate a deeper understanding of its pathogenesis.

## Data Availability Statement

The original contributions presented in the study are included in the article/supplementary material, further inquiries can be directed to the corresponding author.

## Ethics Statement

The studies involving human participants were reviewed and approved by Institutional Review Board of China Medical University. The patients/participants provided their written informed consent to participate in this study.

## Author Contributions

RH: conception and design, acquisition of data, administrative, technical, or material support, and study supervision. RH and XB: development of methodology, analysis and interpretation of data, writing, review, and/or revision of the manuscript. Both authors contributed to the article and approved the submitted version.

## Funding

This study was funded by the Beijing Bethune Charitable Foundation (no. AF-OG-03-1.1-03).

## Conflict of Interest

The authors declare that the research was conducted in the absence of any commercial or financial relationships that could be construed as a potential conflict of interest.

## Publisher's Note

All claims expressed in this article are solely those of the authors and do not necessarily represent those of their affiliated organizations, or those of the publisher, the editors and the reviewers. Any product that may be evaluated in this article, or claim that may be made by its manufacturer, is not guaranteed or endorsed by the publisher.
